# Pesticide Toxicity Footprints of Australian Dietary Choices

**DOI:** 10.3390/nu13124314

**Published:** 2021-11-29

**Authors:** Bradley Ridoutt, Danielle Baird, Javier Navarro, Gilly A. Hendrie

**Affiliations:** 1 Agriculture and Food, Commonwealth Scientific and Industrial Research Organisation (CSIRO), Clayton, VIC 3169, Australia; 2 Department of Agricultural Economics, University of the Free State, Bloemfontein 9300, South Africa; 3 Health and Biosecurity, CSIRO, Adelaide, SA 5000, Australia; danielle.baird@csiro.au (D.B.); gilly.hendrie@csiro.au (G.A.H.); 4 Agriculture and Food, CSIRO, St Lucia, QLD 4067, Australia; javi.navarro@csiro.au

**Keywords:** dietary guidelines, diet quality, discretionary food, ecotoxicity, environment, human toxicity, life cycle assessment, sustainable diet, USEtox model

## Abstract

Pesticides are widely used in food production, yet the potential harm associated with their emission into the environment is rarely considered in the context of sustainable diets. In this study, a life cycle assessment was used to quantify the freshwater ecotoxicity, human toxicity carcinogenic effects, and human toxicity noncarcinogenic effects associated with pesticide use in relation to 9341 individual Australian adult daily diets. The three environmental indicators were also combined into a pesticide toxicity footprint, and a diet quality score was applied to each diet. Energy-dense and nutrient-poor discretionary foods, fruits, and protein-rich foods were the sources of most of the dietary pesticide impacts. Problematically, a dietary shift toward recommended diets was found to increase the pesticide toxicity footprint compared to the current average diet. Using a quadrant analysis, a recommended diet was identified with a 38% lower pesticide toxicity footprint. This was achieved mainly through a reduction in the discretionary food intake and by limiting the choice of fresh fruits. As the latter contradicts dietary recommendations to eat a variety of fruits of different types and colors, we concluded that dietary change may not be the best approach to lowering the environmental impacts of pesticides in the food system. Instead, targeted action in the horticultural industry may be more effective. Consumers might encourage this transition by supporting growers that reduce pesticide use and apply less environmentally harmful active ingredients.

## 1. Introduction

While the primary purpose of dietary guidance is to promote health through the life stages and to reduce the incidence of diet-related disease [1,2], steps are also being taken to integrate environmental sustainability [3,4,5,6]. The food system is a major source of environmental impacts, and the adoption of more sustainable diets [7] is widely viewed as having an important role in contributing to the Sustainable Development Goals [8]. However, the evidence base to support the development of dietary strategies with lower environmental impact is heavily skewed toward greenhouse gas (GHG) emissions [9,10]. Fewer studies have applied reliable environmental indicators to assess the potential of dietary strategies to reduce impacts from using natural resources such as land and water [11,12]. Such impacts are complex to study, as they depend on the local environmental context. For example, water use is of greatest environmental concern in regions that experience water scarcity, not where it is abundant [13]. There are also different types of land use, and regions differ in biodiversity conservation importance [14]. Other environmental concerns, such as the toxicity impacts of pesticide emissions or the human health impacts of fine particulate matter emissions, have rarely been studied in the context of dietary patterns [9]. This means that the evidence base to support sustainable dietary guidance is incomplete and biased toward the reduction in climate impacts.

Pesticide use (insecticides, herbicides, and fungicides) is a present reality across the food production system. Worldwide, it is estimated that around 2 million tons are applied annually [15,16]. These chemicals currently play a vital role in controlling pests and diseases, protecting yields and safeguarding food supplies; however, their emission into the environment is of concern due to their toxicity to humans and the ecosystems and impacts on biodiversity [17,18]. Pesticide emissions are a leading source of surface water pollution [19] and have been linked to losses of up to 40% of stream invertebrate species [20]. The widely used neonicotinoid insecticides are of particular concern, as they are linked to declines in insect populations, including pollinators [21]. Persistent pesticides (i.e., that resist breakdown in the environment) can present long-term risks through accumulation in groundwater and sediments [22]. Human exposure to pesticide pollution can lead to a range of cancers and other diseases [23]. Furthermore, these impacts occur even when pesticides are used within the regulatory limits [19]. Hence, one jurisdiction is producing proactive agricultural policies that aim to substantially reduce the use of pesticides. For example, the European Commission has a target to achieve a 50% reduction in pesticide use and risk by 2030 [24]. The United Nation’s Food and Agriculture Organization also works to improve pesticide management and gives priority to addressing the risks of highly hazardous pesticides [25]. These efforts align with Sustainable Development Goal 12.4 that relates to minimizing the adverse impacts on human health and the environment from chemicals.

It is reasonable to hypothesize that different dietary patterns are associated with lesser or greater environmental impacts from pesticide use, depending on food choices. In the same way that dietary change might be encouraged to support the lowering of GHG emissions across the food system, so too might dietary change be an intervention to support other forms of environmental impact reduction, including impacts from pesticides. It is also important to understand the potential synergies and trade-offs that dietary change might have across multiple environmental aspects. Probably, the reason why so little is known about the variation in pesticide toxicity impacts of diets is due to the sheer complexity of that topic. By one estimate, there are more than 17,000 available pesticides [26] that can broadly be grouped into 40 categories [16]. The use of particular pesticides and amounts varies according to region, crop, and farm management practices. In addition, the absolute mass of the pesticide used is not necessarily a useful indicator of the potential toxicity impact, as individual active ingredients vary in fate, exposure, and effect once emitted into the environment. It is therefore a challenge to integrate this type of information in an environmentally meaningful way across complete diets.

In this study, we apply a life cycle assessment (LCA) to model the pesticide toxicity footprints of 9341 individual Australian adult diets obtained from the Australian Health Survey [27]. These are the same Australian diets that have previously been assessed for climate footprint [28], water scarcity footprint [11], and cropland scarcity footprint [12]. In addition, a diet quality score was calculated, enabling the identification of diets in the Australian community characterized by a higher diet quality and lower pesticide toxicity footprint. The objectives were to understand the variability between Australian diets and the extent to which a dietary change might contribute to lowering pesticide impacts. In addition, we sought to expand the evidence base concerning the environmental impacts of diets in Australia by providing a more complete coverage of the relevant environmental impact categories. As far as we know, this is the first study of its kind to assess the pesticide toxicity footprint associated with diets in a large sample of individuals.

## 2. Materials and Methods

### 2.1. Dietary Intake Data

Dietary intake data were sourced from the nutrition component [29] of the Australian Health Survey [27]. This survey was undertaken by the Australian Bureau of Statistics (ABS) using a complex sampling methodology that enables the application of weighting factors to estimate the dietary intake for the Australian population, as well as demographic subgroups. The data were obtained using a quantitative 24-h recall process, with data collection taking place over a 13-month period to account for variations in eating habits across seasons. Data were also collected across all days of the week. The dataset, covering 9341 Australian adults (19 years and above) and including more than 5000 individual foods and beverages, is considered the most detailed and nationally representative source of dietary intake data in Australia. Nevertheless, as with all 24-h dietary recall studies, there is a potential for the inaccurate recall of foods and portion sizes. To facilitate the use of the dietary intake data, the ABS has published estimates of the prevalence of underreporting of energy (17% for males; 21% for females) [27] that were uniformly applied in this study to avoid the systematic underestimation of dietary pesticide toxicity footprints and to enable reliable comparisons between the reported and recommended diets. The ABS also undertook a second 24-h recall; however, this was only completed by 64% of the original sample, with significantly lower energy intakes reported. In addition, this manuscript describes population estimates rather than the usual intake, and for these reasons, only data from the first recall were used.

ABS population weighting factors were applied to determine the mean dietary intake for Australians, along with mean values for the age and gender subgroups described in the Australian Dietary Guidelines [2]. To enable integration of the dietary data with pesticide toxicity footprint data, composite foods (e.g., sandwich, casserole) were disaggregated into basic components, and cooked foods portions were translated into raw quantities, as described previously [11,12,28]. For each of the 9341 adult daily diets, the total energy intake was determined using the Australian Food Composition Database [30], as well as the number of servings from each food group defined by the Australian Dietary Guidelines [2]. Further subcategorization was undertaken for fresh meats and alternatives, since these foods often feature prominently in discussions about sustainable diets [31,32,33]. Discretionary foods, which are characterized in the Australian Dietary Guidelines [2] as energy-dense and nutrient-poor foods and beverages high in saturated fat, added sugars and salt, and alcohol, were also studied, as they are generally overconsumed in Australia and are a major public health nutrition concern [34,35,36]. Some beverages are included within the prescribed food groups. For example, milk is considered a dairy food, and sugar-sweetened beverages are considered discretionary foods. For other beverages that are not included within the prescribed food groups, such as tea and coffee, a separate group was formed. Tea and coffee beverages were assessed considering the quantity of the tea leaves and coffee beans required, as well as other ingredients added. In summary, foods were classified in a way that was consistent with the contents of the Australian Dietary Guidelines [2], avoiding arbitrary judgements about food classifications.

### 2.2. Diet Quality Analysis

For each of the 9341 individual daily diets, the Diet Quality Index of Golley and Hendrie [37] was used to calculate a diet quality score. These scores, ranging from 0 to 100, describe the level of compliance with the food-based Australian Dietary Guidelines [2], with a higher score reflecting greater compliance.

### 2.3. Pesticide Toxicity Footprint Modelling

Recently, a high-resolution (1.1 km^2^), national-scale map of agricultural pesticide toxicity in Australia was created [38]. The data included 158 chemicals (86 herbicides, 42 insecticides, and 32 fungicides) applied in the production of 25 different crop and livestock commodities. Using these data and parameters from the USEtox impact assessment model [39], the human and ecotoxicological impacts of pesticide use in relation to the production of agricultural commodities in Australia were characterized [38]. The USEtox model was developed under the auspices of the UN Environment’s Life Cycle Initiative [40], and it is widely used in life cycle assessment studies [41]. The model accounts for the fate, exposure, and effect characteristics of thousands of chemicals when emitted into the environment. The agricultural pesticide use mode produced three environmental indicators. The first two were human toxicity carcinogenic and noncarcinogenic effects, both reported in comparative toxicity units (CTUh), where each comparative toxicity unit reflects a potential disease case. The third indicator was freshwater ecotoxicity, also expressed in comparative toxicity units (CTUe), reflecting potential species loss. The details of this model are available in the associated references [38,39].

Australia is a net exporter of most agricultural commodities, and it is estimated that more than 90% of the food available to Australian consumers is produced locally [42,43]. As such, the data resource described above provided a broad coverage of most Australian foods. For items not produced in Australia to any significant extent, namely tea leaves, coffee beans, hops, palm fruit, cocoa beans and coconut, the method described above [38] was replicated to provide equivalent data.

To facilitate understanding of the combined human and ecotoxicological impacts of pesticide use, a fourth indicator, pesticide toxicity footprint, was also developed. To calculate the pesticide toxicity footprint, the individual USEtox results were scaled relative to estimates of the total global human and ecotoxicological burden of chemicals [44] and summed using equal weighting. The choice to equally weight the indicator results is a value judgement that implies equal importance of the three types of environmental impacts. For the sensitivity analysis, two alternative footprint quantification models were developed. The first alternative scaled the individual footprint results relative to estimates of the total burden in the EU27 countries [45] and then applied equal weighting. The second alternative scaled the individual footprint results relative to the total global burden (i.e., the same as the base calculation) but then summed the indicator results using weighting factors reflecting the relative importance expressed in EU policy targets [46]. Global and EU references were used in the absence of equivalent data for Australia. Normalization and weighting factors are reported in Supplementary Table S1.

The pesticide toxicity indicator results for individual foods were quantified using conversion factors that translate agricultural commodities into retail products and edible portions, as described previously [11,12]. A complete list of the results for foods used in this study is presented in Supplementary Table S2.

### 2.4. Dietary Patten Model

Environmental indicators were calculated for each of the 9341 individual adult daily diets. In addition, for the largest adult subgroup, the 19–50-year age group (*n* = 5157), quadrant analyses were performed to identify diets with higher diet quality and lower pesticide toxicity impacts. Separate quadrant analyses were performed for each environmental indicator. In performing the quadrant analyses, daily diets within 0.25 standard deviations of the mean of each parameter were excluded to achieve greater contrast. Secondly, a dietary pattern was modeled by scaling the average daily diet of the 19–50-year age grouping so that the recommended number of servings of each food group were achieved, based on the Australian Dietary Guidelines [2]. The premise here is that individuals eat the same types of fruits, vegetables, cereal foods, discretionary foods, etc. but in the recommended proportions. The Australian Dietary Guidelines [2] are flexible and, while emphasizing the importance of variety, do not prescribe particular food choices. Therefore, many ways of complying with the guidelines are possible. Thirdly, a recommended diet was modeled based on the food choices evident in the higher diet quality and lower pesticide toxicity impact quadrant described above. As such, the dietary patterns that were assessed were identified from within the observed data, meaning they were consistent with the population food preferences. No attempt was made to construct dietary patterns based on arbitrary food substitutions defined by the authors, which could potentially introduce bias into the research design.

### 2.5. Correlation Analysis

As the data were continuous and in related pairs, the Pearson correlation coefficient was used to evaluate the relationships between results obtained using the different pesticide toxicity indicators (*n* = 9341), as well as between the dietary energy intake and pesticide toxicity footprint (*n* = 9341). Furthermore, correlations between the dietary pesticide toxicity footprint and other environmental footprints (climate footprint, water-scarcity footprint, and cropland scarcity footprint) were explored using data from previous studies [11,12,28] (*n* = 9341). Correlations between footprints were undertaken after controlling for variations in the dietary energy content.

## 3. Results

### 3.1. Pesticide Toxicity Footprint and Energy Intake

**Figure 1:**
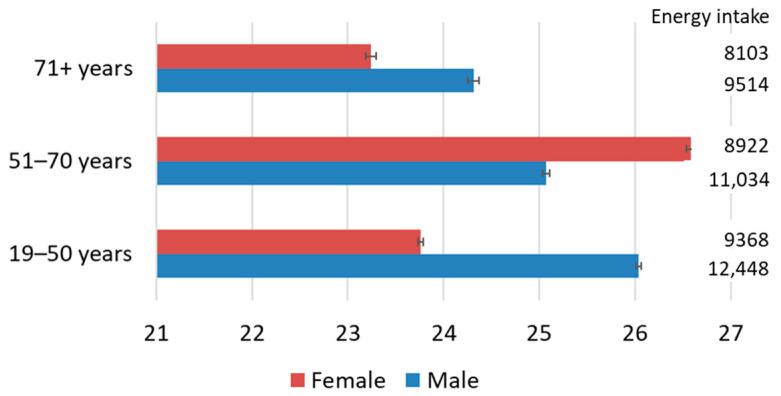
The pesticide toxicity footprint (points) and energy intake (kJ day^−1^) of Australian adult diets based on 9341 individual daily diets reported in the Australian Health Survey. Bars show 95% confidence intervals.

The pesticide toxicity footprint of Australian adult daily diets averaged 25.1 points per person (95% CI 24.6–25.5). The score was lowest for older females, averaging 23.2 points per person, and highest for females in the 51–70 age group, where the score averaged 26.6 points per person (Figure 1). The pesticide toxicity footprint and total dietary energy intake were positively correlated. However, variations in the total energy intake explained little of the variations in the pesticide toxicity footprint (*R^2^* = 0.1) in contrast to previous studies, where variations in the total dietary energy intake explained around 45% of the variations in the dietary cropland scarcity footprint [12], around one-third of the variations in the dietary water scarcity footprint [11], and around one-quarter of the variations in the dietary climate footprint [28]. As such, the dietary pesticide toxicity footprints appeared to be primarily determined by food choice. The pesticide toxicity footprint aggregates the human and ecotoxicological impacts of pesticide use. For the average Australian adult diet, the individual results for freshwater ecotoxicity, human toxicity carcinogenic effects, and human toxicity noncarcinogenic effects were 4.0 CTEe, 1.09 × 10^−9^ CTUh, and 1.64 × 10^−8^ CTUh per person (Supplementary Figure S1). Regarding the alternative pesticide toxicity footprint weighting models, the results obtained were highly correlated with the base model (*r* > 0.999, *p* < 0.01). As such, the results obtained with these alternative models are not further discussed.

### 3.2. Contribution Analysis

The pesticide toxicity footprint results were disaggregated based on the food groupings described in the Australian Dietary Guidelines [2]. The largest contribution came from discretionary foods at around 30% (Table 1), closely followed by fruits at around 28%. Discretionary foods are non-core foods that are often energy-dense but contribute few beneficial nutrients. They are recommended to be eaten only occasionally and in small quantities [2]. According to the Australian Dietary Guidelines, fresh and dried fruits, as well as fruit juice that is prepared without an added sweetener, are all considered servings of fruits. However, fruits that may be incorporated into pastries, muesli bars, sweetened beverages, and the like are not included, as these foods are classified as discretionary foods. The Australian Dietary Guidelines [2] group together meats and other protein-rich alternatives such as eggs, nuts, and legumes. Together, these foods made the third-largest contribution to the pesticide toxicity footprint at around 18%. Within this food group, poultry made the largest contribution (6.6%), followed by red meat (5.4%) and vegetarian alternatives (4.4%). Other food groups made lesser contributions in the order of 5% or less (Table 1).

Considering the individual pesticide toxicity indicators, the contribution of discretionary foods remained high (28.2–33.4%, Table 1). The contribution of fruits was also high for the freshwater ecotoxicity and human toxicity noncarcinogenic effects (29.2 and 19.6%, respectively) but lower for the human toxicity carcinogenic effects (9.3%). In the case of human toxicity carcinogenic effects, the contribution of livestock products was higher, e.g., poultry (11.2%), dairy and alternatives (10.2%), and red meat (9.6%; Table 1). These differences are explained by the particular combinations of pesticides used in each agricultural industry and their diverse toxicological effects [38].

### 3.3. Dietary Pattern Analysis

**Figure 2:**
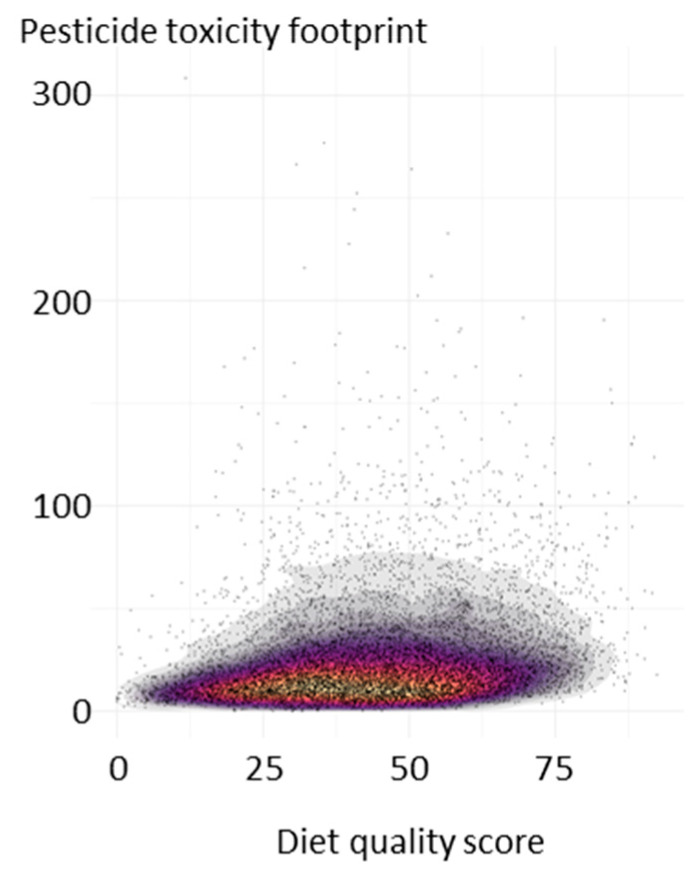
Pesticide toxicity footprint (points) and diet quality score (range 0–100). Scatterplot showing the diversity in the adult daily diets reported in the Australian Health Survey (*n* = 9341). Shading indicates the density of the points from high (yellow) to low (grey).

Individual daily diets varied greatly in both pesticide toxicity footprint and diet quality score (Figure 2), with only a weak positive correlation between the two dimensions (Pearson correlation: 0.21). Considering the largest age group (19–50 years, *n* = 5157), the current average diet was compared to the higher diet quality and lower pesticide toxicity footprint subgroup identified by the quadrant analysis. This subgroup had a 42% higher diet quality score (58.3 compared to 41.0) and a 53% lower pesticide toxicity footprint (11.7 compared to 24.9; Table 2). Compared to the average diet, this subgroup had a greatly reduced intake of discretionary foods (2.31 servings compared to 7.42 servings). There was also an increased intake of most core foods, especially vegetables (3.61 servings compared to 2.47 servings), seafood (0.32 servings compared to 0.22 servings), and cereal foods. The reduced intake of discretionary foods contributed around 30% of the reduction in the pesticide toxicity footprint. However, the largest reduction in the pesticide toxicity footprint came from fruits (around 64%) and predominantly through the choice of fruits generally with a lower pesticide toxicity footprint (e.g., apples and pears). The total intake of fruits varied marginally (1.22 servings compared to 1.38 servings for the current average diet; Table 2).

**Figure 3:**
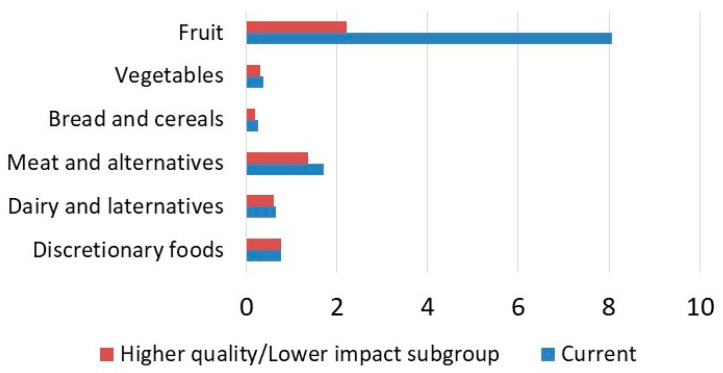
Pesticide toxicity footprint intensity of the major food groups (points per serving). Shown are the results for the current average 19−50-year-old Australian daily diet, as well as a higher diet quality and lower pesticide toxicity footprint subgroup identified by the quadrant analysis.

A recommended diet based on the Australian Dietary Guidelines [2] was also constructed for 19–50-year-old adults by scaling the average 19–50-year-old diet (Table 3). This involved scaling upward the intake of core foods. For example, the intake of fruits was increased from 1.38 servings in the current average diet to the recommended two servings. Meat and alternatives were scaled upward but only slightly from 2.32 to 2.8 servings and in proportion to the current pattern of intakes of seafood, red meat, poultry, etc. The intake of discretionary food was scaled downward. This process resulted in a recommended diet with a pesticide toxicity footprint of 29.40 points per person, an increase of almost 18%, underscoring the fact that healthy diets do not necessarily have lower environmental impacts. However, if the dietary pattern evident in the higher diet quality and lower pesticide toxicity footprint subgroup was scaled to conform with the recommended number of servings described in the Australian Dietary Guidelines, the pesticide toxicity footprint became 15.35 points per person (Table 3), 38% lower than the current average diet. Again, most of the reduction in the pesticide toxicity footprint came from the reduced intake of discretionary foods and the inclusion of fruits with a lower pesticide toxicity footprint. As shown in Figure 3, the variations in the pesticide footprint intensity (points per serving) were exceptionally large for fruits. Tropical stone fruits were determined to have a pesticide toxicity footprint (per kg edible portion) more than twice that of citrus fruits and more than 50 times that of some pip fruits (Supplementary Table S2).

## 4. Discussion

### 4.1. Comparison with Other Studies

Pesticides are commonly used during food production; yet, the potential harm caused from their emission into the environment is seldom considered in the context of sustainable diets [9], despite their use being a long-term concern [47]. As an example, the reduction in harm from pesticides use was not considered in the development of the widely discussed EAT-Lancet planetary health diet [33]. A lack of adequate data has been identified as one of the contributing reasons [48,49,50,51]. As such, the pesticide toxicity footprints quantified for individual Australian adult diets in this study contributed important new evidence to inform the transition to sustainable diets. It is not easy to find comparable results in the literature. Marlow et al. [52] quantified the mass of pesticide active ingredients used in the production of foods for diets surveyed during the Adventist Health Survey in California. A dietary pesticide demand of 3.7–4.5 g of active ingredient per day were reported. However, by assessing only the mass of active ingredients, the fate, exposure, and effect characteristics of each pesticide were not considered. This is a limitation, since one strategy to reduce the harm from pesticide use in agriculture is to use pesticides that are equally effective but pose less risk to the environment. Such a strategy, while reducing potential environmental harm, might not involve an absolute decrease in the active ingredient. Castellani et al. [46] assessed a basket of 17 food products representative of EU27 consumption determined from food expenditure data using LCA. Human and ecotoxicological impacts relating to pesticides and other chemical emissions were identified as an important environmental impact category. Most studies addressing the environment impacts of pesticides address individual food products, such as citrus [53], strawberries [54,55], cherries [56], pineapples [57], or other crops [58], not entire diets. In this study of Australian adult daily diets, it was shown that the human and ecotoxicological impacts relating to pesticide use vary widely between individual diets and that these potential impacts are determined largely by food choice. The three main types of foods contributing to these impacts were discretionary foods, fruits, and protein-rich foods, which will each be discussed next.

### 4.2. Role of Discretionary Foods

Energy-dense and nutrient-poor foods that are high in saturated fat, added sugars, and salt are a part of the Australian food system. According to the Australian Dietary Guidelines, these foods, as well as alcohol, should only be consumed sometimes and in small amounts [2]. However, in general, Australian adults consume more than twice the recommended servings of these foods (Table 2) at the expense of healthy core foods (Table 3). On average, Australians fail to reach the recommended intake of any of the five food groups (Table 3). Therefore, not surprisingly, discretionary foods made the largest contribution to the pesticide toxicity footprint and contributed around 30% of the individual pesticide toxicity indicator scores (Table 1). This is consistent with the results reported for dietary water scarcity, cropland scarcity, and climate footprints in Australia [11,12,28], and the importance of limiting discretionary foods as part of a sustainable diet has also been noted elsewhere [59,60]. The lack of overt consideration of these types of foods in many sustainable diet studies and recommendations is clearly a major oversight that needs addressing. However, it is important to note that, while reducing the intake of discretionary foods and increasing the intake of core foods will greatly improve a diet’s quality, it will not necessarily lead to a lower pesticide toxicity footprint. A recommended diet based on the current food choices was found to have an 18% higher pesticide toxicity footprint (24.93 and 29.40 points per person per day; Table 2 and Table 3, respectively). Discretionary foods make the largest contribution to the pesticide toxicity footprint of Australian adult diets, because these foods are so abundant in the average Australian diet, not because individually they have an exceptionally high pesticide toxicity footprint.

### 4.3. Role of Fruits

Fruits also make a large contribution to the dietary pesticide toxicity footprint (28.3%; Table 1), which is of concern, because Australian adults generally consume less than the recommended amount of fruits (1.38 servings per person per day compared to the recommendation of two servings; Table 2) and are encouraged to consume more [2]. Increasing the intake of fruits has the potential to dramatically increase the dietary pesticide toxicity footprint (Table 3). This evidence relating to fruits highlights the issue that higher-quality diets do not necessarily have lower environmental impacts. In the UK, increasing the content of fruits and vegetables in the diet was found to increase greenhouse gas emissions [61]. In Denmark, vegetarian and vegan diets were reported to have water scarcity footprints 26% and 31% higher than the average diet [62]. In Sweden, Moberg et al. [50] identified the potential for the increased consumption of plant-based foods to increase the pesticide use and other environmental impacts. Affordability impacts have also been shown, whereby pathways to increased fruit and vegetable intakes in the UK led to higher diet costs [63]. Sustainability is ultimately about trade-offs and about finding solutions where there are competing objectives. In Australia, a higher intake of fruits is possible without increasing the pesticide toxicity footprints, as demonstrated by the recommended diet based on lower-impact food choices (Table 3). In Australia, different fruits vary widely in pesticide toxicity footprints (Figure 3 and Table S2). However, it may be counterproductive to recommend a higher fruit consumption while also suggesting that the choice of fruits should be limited to reduce the pesticide toxicity impacts. This would contradict the important guideline of seeking variety in fruit and vegetable intakes [2]. An alternative solution is action in the horticultural industry to reduce the quantity of pesticide used and to transition to active ingredients with lower toxicity impacts in the environment. Environmental labeling schemes to inform consumers could also be investigated.

### 4.4. Role of Protein Foods

Dietary strategies in the academic literature that aim to lower environmental impacts often emphasize a transition away from livestock products, especially ruminant meat and dairy products [64,65,66]. In Australia, the meat and alternatives food group contributed less to the total pesticide toxicity footprint than discretionary foods and fruits but was the next highest (18.3%; Table 1). Interestingly, compared to the current average adult diet, those diets in the higher diet quality and lower impact subgroup had 15% higher intakes of red meat (0.76 servings per person per day compared to 0.66) and marginally higher intakes of dairy foods (Table 2). In Australia, ruminant meats were determined to have pesticide toxicity footprints of 1.01 points per 90 g of serving for beef and 1.15 points per 90 g of serving for lamb, which are lower than the scores attributed to chicken (1.71 points per 100 g of serving) and pork (2.24 points per 90 g of serving) (see Table S2 for details). This is consistent with the ecotoxicity results presented by Nordborg et al. [67], whereby chicken fillets and minced pork had larger impacts than minced beef. In Australia, some vegetarian alternatives had very low pesticide toxicity footprints (e.g., tofu, 170 g per serving, 0.19 points), but others were assessed as having higher impacts (e.g., legumes, 150 g soaked, 1.62 points). The main point here is that it is difficult to more broadly generalize about plant-based and animal-sourced protein-rich foods in terms of their pesticide footprint and environmental impacts.

### 4.5. Implications for Other Environmental Aspects

Environmental sustainability is a complex subject due to the many environmental aspects [9], and it can be a challenge to achieve multiple environmental improvement objectives simultaneously, because individual foods have different impacts on the environment. For example, in this study, fruits had the highest pesticide toxicity footprint scores per serving (Figure 3), and although citrus and summer stone fruits grown in Australia can also have high water scarcity footprints [11], fruits tend to have a low climate footprint and low cropland demand due to their high productivity [68]. The dietary pesticide toxicity footprints (*n* = 9341) were moderately correlated with the dietary water scarcity footprints (*r* = 0.66, *p* < 0.01) but only very weakly correlated with cropland scarcity footprints (*r* = 0.22, *p* < 0.01) and not correlated with greenhouse gas emissions. In previous studies, it was found that efforts to reduce the climate impacts of Australian adult diets through dietary changes resulted in increases in water scarcity impacts [69]. The question as to whether certain environmental objectives should take precedence over others is controversial and subject to value judgements that can differ from one individual or group to another. As such, care is needed in evaluating dietary change recommendations that are designed to achieve improvement in one environmental dimension only. However, it is suggested that, in the future, pesticide impacts should be combined with other environmental metrics, so that the environmental impacts of diets are studied as completely as possible and trade-offs between environmental impact categories are understood and managed.

### 4.6. Limitations

The evidence presented in this article is based on Australian dietary intake data and pesticide toxicity impact assessments for foods that are produced and available in Australia. As dietary patterns and food production practices differ across regions, the results may not be applicable in other geographical contexts. The pesticide impacts were assessed for emissions to the environment via agricultural soils. We lacked data for the pesticide spray drift, which could have led to an underestimation of the impacts associated with horticultural products, since they are often grown in closer proximity to water bodies and urban areas than crops and pastures [38]. The model did not include potential human health impacts relating to exposure to pesticides during food consumption (i.e., through residues) [70], as this is very dependent on individual food handling and preparation practices that were not assessed during the dietary intake survey. Nor did the study consider potential human exposure to chemicals in food packaging [71]. As already mentioned, environmental sustainability is a complex subject with many different environmental aspects, and the results reported for the pesticide toxicity footprint could not be used to make conclusions about the overall level of environmental sustainability.

Care was taken to use the highest quality dietary intake data. However, all data obtained by 24-h dietary recall is potentially subject to inaccurate reporting. Fruits and vegetables may be overreported in dietary surveys, and therefore, it is possible that the contribution of these foods has been overestimated. Conversely, discretionary foods are more likely to be underreported; therefore, it is possible that the contribution of these foods was underestimated. However, this would not change the conclusions, since discretionary foods were already identified as the highest contributing food group to the total dietary pesticide toxicity footprint. To calculate the pesticide toxicity footprint, there is subjectivity in the choices of normalization and the weighting factors. However, a sensitivity analysis was undertaken using alternative factors, and the results were consistent.

## 5. Conclusions

The environmental impacts of pesticide emissions during food production are a relevant environmental concern, and this study provided new understanding about the variations among individual daily diets and the main contributing food groups, namely discretionary foods, fruits, and meats and alternatives. Problematically, a dietary shift toward recommended diets was found to increase the pesticide toxicity footprint compared to the current average diet. However, by the quadrant analysis, a recommended diet with a lower pesticide toxicity footprint was also identified. This was achieved mainly through limiting the choice of fresh fruits. We concluded that dietary change may not be the best approach to lowering the environmental impacts of pesticides in the food system. Instead, targeted action in the horticultural industry may be more effective. Consumers might encourage this transition by favoring products from growers that employ practices that minimize the use of pesticides and, especially, the use of active ingredients with high levels of toxicity when emissions into the environment occur.

## Figures and Tables

**Table 1:** Freshwater ecotoxicity (FE), human toxicity carcinogenic effects (HT-c), human toxicity noncarcinogenic effects (HT-nc), and pesticide toxicity footprint (PTF). Food groups are as defined by the Australian Dietary Guidelines.

Food	FE	HT-c	HT-nc	PTF
Fruit	29.2	9.3	19.6	28.3
Vegetables	5.5	8.6	4.8	5.5
Breads and cereals	6.0	11.6	12.9	6.6
Meat and alternatives	17.6	29.6	21.8	18.3
Seafood	(0.3)	(0.9)	(0.5)	(0.3)
Red Meat	(5.2)	(9.6)	(6.7)	(5.4)
Poultry	(6.3)	(11.2)	(8.1)	(6.6)
Vegetarian alternatives (eggs, etc.)	(4.4)	(5.2)	(4.3)	(4.4)
Reptiles, offal	(<0.1)	(<0.1)	(<0.1)	(<0.1)
Pork	(1.4)	(2.5)	(2.2)	(1.5)
Dairy and alternatives	5.6	10.2	5.3	5.7
Beverages	3.2	0.6	0.3	2.9
Discretionary foods	29.8	28.2	33.4	29.7
Sugar-sweetened beverages	(2.4)	(0.3)	(1.4)	(2.2)
Savory and sweet biscuits, cakes, and waffles	(2.9)	(2.9)	(2.9)	(2.9)
Sweet and savory pastries, pies	(1.4)	(2.5)	(1.7)	(1.5)
Processed meat, burgers, tacos, pizza, and hot dogs	(6.7)	(12.1)	(9.6)	(7.1)
Dairy based desserts, cream, and butter	(1.4)	(2.0)	(1.1)	(1.4)
Fried potato products and extruded snacks	(2.0)	(2.4)	(1.8)	(2.0)
Muesli bars, confectionary, and chocolate	(4.0)	(1.5)	(2.7)	(3.8)
Alcohol	(7.2)	(2.5)	(10.5)	(7.2)
Other	(1.8)	(2.0)	(1.6)	(1.8)
Healthy fats and oils	2.7	1.1	1.6	2.6
Miscellaneous	0.4	0.6	0.4	0.4

**Table 2:** Food intake (servings person^−1^) and pesticide toxicity footprint (PTF, points per person^−1^) for the average daily diet of 19–50-year-old Australian adults (*n* = 5157) and a higher diet quality and lower pesticide toxicity footprint subgroup (*n* = 1677) identified by the quadrant analysis. See the text for details. Food groups are as defined by the Australian Dietary Guidelines.

Food	Current		Higher Diet Quality and Lower Impact Subgroup	
	Servings	PTF	Servings	PTF
Fruit	1.38	11.14	1.22	2.72
Vegetables	2.47	0.92	3.61	1.09
Breads and cereals	4.57	1.17	5.98	1.09
Meat and alternatives	2.32	3.98	2.55	3.45
Seafood	(0.22)	(0.06)	(0.32)	(0.06)
Red Meat	(0.66)	(0.87)	(0.76)	(0.99)
Poultry	(0.74)	(1.44)	(0.78)	(1.47)
Vegetarian alternatives	(0.51)	(1.29)	(0.49)	(0.60)
Reptiles, offal	(<0.01)	(<0.01)	(<0.01)	(<0.01)
Pork	(0.18)	(0.32)	(0.19)	(0.33)
Dairy and alternatives	1.46	0.97	1.50	0.89
Discretionary foods	7.42	5.66	2.31	1.76
Miscellaneous		1.08		0.73
Total		24.93		11.74

**Table 3:** Food intake (servings person^−1^) and pesticide toxicity footprint (PTF, points per person^−1^) for recommended diets for 19–50-year-old Australian adults. Recommended diets are based on the food choices evident in the current average diet (*n* = 5175) and the lower pesticide toxicity footprint subgroup (*n* = 1677) identified by the quadrant analysis. See the text for details. Food groups are as defined by the Australian Dietary Guidelines.

Food	Recommended Diet Based on Average Food Choices		Recommended Diet Based on Lower Pesticide Toxicity Footprint Food Choices	
	Servings	PTF	Servings	PTF
Fruit	2.0	16.12	2.0	4.44
Vegetables	5.5	2.05	5.5	1.66
Breads and cereals	6.0	1.54	6.0	1.10
Meat and alternatives	2.8	4.81	2.8	3.79
Seafood	(0.27)	(0.07)	(0.35)	(0.07)
Red Meat	(0.79)	(1.05)	(0.83)	(1.09)
Poultry	(0.90)	(1.74)	(0.86)	(1.62)
Vegetarian alternatives	(0.61)	(1.56)	(0.54)	(0.66)
Reptiles, offal	(<0.01)	(<0.01)	(<0.01)	(<0.01))
Pork	(0.22)	(0.38)	(0.21)	(0.36)
Dairy and alternatives	2.5	1.66	2.5	1.49
Discretionary foods	2.8	2.14	2.8	2.14
Miscellaneous		1.08		0.73
Total		29.40		15.35

## Data Availability

The dietary intake data are available from the Australian Bureau of Statistics (http://www.abs.gov.au/australianhealthsurvey, accessed on 15 March 2017). The pesticide toxicity footprints of individual foods are presented in Supplementary Table S2.

## Supplementary Materials

The following are available online at https://www.mdpi.com/article/10.3390/nu13124314/s1. Table S1: Normalization and weighting factors. Table S2: Environmental indicator values of individual foods. Figure S1: Environmental indicator results for Australian adult daily diets.

## References

[1] USDA (2020). Dietary Guidelines for Americans 2020–2025.

[2] Australia Government (2013). Australian Dietary Guidelines Summary.

[3] Fischer C.G., Garnett T. (2016). Plates, Pyramids, Planet, Developments in National Healthy and Sustainable Dietary Guidelines: A State of Play Assessment.

[4] Swedish National Food Agency (2015). The Swedish Dietary Guidelines.

[5] Public Health England (2018). The Eatwell Guide.

[6] Nordic Council of Ministers (2014). Nordic Nutrition Recommendations 2012, Integrating Nutrition and Physical Activity.

[7] Drewnowski A., Finley J., Hess J.M. (2020). Towards healthy diets from sustainable food systems. Curr. Dev. Nutr..

[8] United Nations (2015). Transforming Our World: The 2030 Agenda for Sustainable Development.

[9] Ridoutt B.G., Hendrie G.A., Noakes M. (2017). Dietary strategies to reduce environmental impact: A critical review of the evidence base. Adv. Nutr..

[10] Jones A.D., Hoey L., Blesh J., Miller L., Green A., Shapiro L.F. (2016). A systematic review of the measurement of sustainable diets. Adv. Nutr..

[11] Ridoutt B.G., Baird D., Anastasiou K., Hendrie G.A. (2019). Diet quality and water scarcity: Evidence from a large Australian population health survey. Nutrients.

[12] Ridoutt B., Anastasiou K., Baird D., Navarro Garcia J., Hendrie G. (2020). Cropland footprints of Australian dietary choices. Nutrients.

[13] Ridoutt B.G., Huang J. (2012). Environmental relevance—The key to understanding water footprints. Proc. Natl. Acad. Sci. USA.

[14] Chaudhary A., Brookes T.M. (2018). Land use intensity-specific global characterization factors to assess product biodiversity footprints. Environ. Sci. Technol..

[15] Sharma A., Kumar V., Shahzad B., Tanveer M., Sidhu G.P., Handa N., Kohli S.K., Yadav P., Bali A.S., Parihar R.D. (2019). Worldwide pesticide usage and its impacts on ecosystem. SN Appl. Sci..

[16] Zhang W.J. (2018). Global pesticide use: Profile, trend, cost / benefit and more. Proc. Int. Acad. Ecol. Environ. Sci..

[17] Choudri B.S., Charabi Y., Al-Nasiri N., Al-Awadhi T. (2020). Pesticides and herbicides. Water Environ. Res..

[18] Bonmatin J.-M., Giorio C., Sánchez-Bayo F., van Lexmond M.B. (2021). An update of the Worldwide Integrated Assessment (WIA) on systemic insecticides. Environ. Sci. Pollut. Res..

[19] Stehle S., Schulz R. (2015). Agricultural insecticides threaten surface waters at the global scale. Proc. Natl. Acad. Sci. USA.

[20] Beketov M.A., Kefford B.J., Schäfer R.B., Liess M. (2013). Pesticides reduce regional biodiversity of stream invertebrates. Proc. Natl. Acad. Sci. USA.

[21] Kavanagh S., Henry M., Stout J.C., White B. (2021). Neonicotinoid residues in honey from urban and rural environments. Environ. Sci. Pollut. Res..

[22] McKnight U.S., Rasmussen J.J., Kronvang B., Binning P.J., Bjerg P.L. (2015). Sources, occurrence and predicted aquatic impact of legacy and contemporary pesticides in streams. Environ. Pollut..

[23] Kim K.-H., Kabir E., Jahan S.A. (2017). Exposure to pesticides and the associated human health effects. Sci. Total Environ..

[24] European Commission, Farm to Fork Targets—Progress. https://ec.europa.eu/food/plants/pesticides/sustainable-use-pesticides/farm-fork-targets-progress_en.

[25] Food and Agriculture Organization of the United Nations, Q&A on Pests and Pesticide Management. http://www.fao.org/news/story/en/item/1398779/icode/.

[26] Pesticide Action Network, Pesticides 101. https://www.panna.org/pesticides-big-picture/pesticides-101.

[27] Australian Bureau of Statistics (2017). 4363.0—National Health Survey: Users’ Guide, 2014–2015.

[28] Ridoutt B., Baird D., Hendrie G.A. (2021). Diets within environmental limits: The climate impact of current and recommended Australian diets. Nutrients.

[29] Australian Bureau of Statistics (2014). 4364.0.55.007—Australian Health Survey: Nutrition First Results—Foods and Nutrients, 2011–2012.

[30] Food Standards Australia New Zealand Australian Food Composition Database. https://www.foodstandards.gov.au/science/monitoringnutrients/afcd/Pages/default.aspx.

[31] Macdiarmid J.L., Douglas F., Campbell J. (2016). Eating like there’s no tomorrow: Public awareness of the environmental impact of food and reluctance to eat less meat as part of a sustainable diet. Appetite.

[32] Stubbs R.J., Scott S.E., Duarte C. (2018). Responding to food, environment and health challenges by changing meat consumption behaviours in consumers. Nutr. Bull..

[33] Willett W., Rockström J., Loken B., Springmann M., Lang T., Vermeulen S., Garnett T., Tilman D., DeClerck F., Wood A. (2019). Food in the Anthropocene: The EAT-Lancet Commission on healthy diets from sustainable food systems. Lancet.

[34] Lal A., Peeters A., Brown V., Nguyen P., Tran H.N.Q., Nguyen T., Tonmukayakul U., Sacks G., Calache H., Martin J. (2020). The modelled population obesity-related health benefits of reducing consumption of discretionary foods in Australia. Nutrients.

[35] Fayet-Moore F., McConnell A., Cassettari T., Petocz P. (2019). Breakfast choice is associated with nutrient, food group and discretionary intakes in Australian adults at both breakfast and the rest of the day. Nutrients.

[36] Zheng M., Rangan A., Meertens B., Wu J.H.Y. (2017). Changes in typical portion sizes of commonly consumed discretionary foods among Australian adults from 1995 to 2011–2012. Nutrients.

[37] Golley R.K., Hendrie G.A. (2014). The Dietary Guidelines Index for children and adolescents: What is the impact of the new dietary guidelines?. Nutr. Diet..

[38] Navarro J., Hadjikakou M., Ridoutt B., Parry H., Bryan B.A. (2021). Pesticide toxicity hazard of agriculture: Regional and commodity hotspots in Australia. Environ. Sci. Technol..

[39] Rosenbaum R.K., Bachmann T., Gold L.S., Huijbregts M., Jolliet O., Juraske R., Koehler A., Larsen H.F., MacLeod M., Margni M. (2008). USEtox–the UNEP-SETAC toxicity model: Recommended characterisation factors for human toxicity and freshwater ecotoxicity in life cycle impact assessment. Int. J. Life Cycle Assess..

[40] Life Cycle Initiative. https://www.lifecycleinitiative.org/.

[41] Finnveden G., Hauschild M.Z., Ekvall T., Guinée J., Heijungs R., Hellweg S., Koehler A., Pennington D., Suh S. (2009). Recent developments in Life Cycle Assessment. J. Environ. Manag..

[42] Ridoutt B., Baird D., Bastiaans K., Darnell R., Hendrie G., Riley M., Sanguansri P., Syrette J., Noakes M., Keating B. (2017). Australia’s nutritional food balance: Situation, outlook and policy implications. Food Sec..

[43] The Prime Minister’s Science, Engineering and Innovation Council (2010). Australia and Food Security in a Changing World.

[44] Crenna E., Secchi M., Benini L., Sala S. (2019). Global environmental impacts: Data sources and methodological choices for calculating normalization factors for LCA. Int. J. Life Cycle Assess..

[45] Sala S., Benini L., Mancini L., Pant R. (2015). Integrated assessment of environmental impact of Europe in 2010: Data sources and extrapolation strategies for calculating normalisation factors. Int. J. Life Cycle Assess..

[46] Castellani V., Sala S., Benini L. (2017). Hotspots analysis and critical interpretation of food life cycle assessment studies for selecting eco-innovation options and for policy support. J. Clean. Prod..

[47] Margni M., Rossier D., Crettaz P., Jolliet O. (2002). Life cycle impact assessment of pesticides on human health and ecosystems. Agric. Ecosyst. Environ..

[48] Tidåker P., Potter H.K., Carlsson G., Röös E. (2021). Towards sustainable consumption of legumes: How origin, processing and transport affect the environmental impact of pulses. Sustain. Prod. Consum..

[49] Notarnicola B., Tassielli G., Renzulli P.A., Castellani V., Sala S. (2017). Environmental impacts of food consumption in Europe. J. Clean. Prod..

[50] Moberg E., Säll S., Hansson P.-A., Röös E. (2021). Taxing food consumption to reduce environmental impacts—Identification of synergies and goal conflicts. Food Policy.

[51] Beylot A., Secchi M., Cerutti A., Merciai S., Schmidt J., Sala S. (2019). Assessing the environmental impacts of EU consumption at macro-scale. J. Clean. Prod..

[52] Marlow H.J., Harwatt H., Soret S., Sabaté J. (2015). Comparing the water, energy, pesticide and fertilizer usage for the production of foods consumed by different dietary types in California. Public Health Nutr..

[53] Soheilifard F., Marzban A., Raini M.G., Taki M., van Zelm R. (2020). Chemical footprint of pesticides used in citrus orchards based on canopy deposition and off-target losses. Sci. Total Environ..

[54] Soode-Schimonsky E., Richter K., Weber-Blaschke G. (2017). Product environmental footprint of strawberries: Case studies in Estonia and Germany. J. Environ. Manag..

[55] Romero-Gámez M., Suárez-Rey E.M. (2020). Environmental footprint of cultivating strawberry in Spain. Int. J. Life Cycle Assess..

[56] Tassielli G., Notarnicola B., Renzulli P.A., Arcese G. (2018). Environmental life cycle assessment of fresh and processed sweet cherries in southern Italy. J. Clean. Prod..

[57] Ingwersen W.W. (2012). Life cycle assessment of fresh pineapple from Costa Rica. J. Clean. Prod..

[58] Peña N., Knudsen M.T., Fantke P., Antón A., Hermansen J.E. (2019). Freshwater ecotoxicity assessment of pesticide use in crop production: Testing the influence of modeling choices. J. Clean. Prod..

[59] Hallström E., Håkansson N., Åkesson A., Wolk A., Sonesson U. (2018). Climate impact of alcohol consumption in Sweden. J. Clean. Prod..

[60] Perignon M., Sinfort C., El Ati J., Traissac P., Drogué S., Darmon N., Amiot M.J., Achir N., Alouane L., Bellagha S. (2019). How to meet nutritional recommendations and reduce diet environmental impact in the Mediterranean region? An optimization study to identify more sustainable diets in Tunisia. Glob. Food Sec..

[61] Dogbe W., Revoredo-Giha C. (2021). Nutritional and environmental assessment of increasing the content of fruit and vegetables in the UK diet. Sustainability.

[62] Goldstein B., Hansen S.F., Gjerris M., Laurent A., Birkved M. (2016). Ethical aspects of life cycle assessments of diets. Food Policy.

[63] Colombo P.E., Milner J., Scheelbeek P.F.D., Taylor A., Parlesak A., Kastner T., Nicholas O., Elinder L.S., Dangour A.D., Green R. (2021). Pathways to “5-a-day”: Modeling the health impacts and environmental footprints of meeting the target for fruit and vegetable intake in the United Kingdom. Am. J. Clin. Nutr..

[64] Garnett T. (2016). Plating up solutions. Science.

[65] Godfray H.C.J., Aveyard P., Garnett T., Hall J.W., Key T.J., Lorimer J., Pierrehumbert R.T., Scarborough P., Springmann M., Jebb S.A. (2018). Meat consumption, health, and the environment. Science.

[66] Aleksandrowicz L., Green R., Joy E.J.M., Smith P., Haines A. (2016). The impacts of dietary change on greenhouse gas emissions, land use, water use, and health: A systematic review. PLoS ONE.

[67] Nordborg M., Davis J., Cederberg C., Woodhouse A. (2017). Freshwater ecotoxicity impacts from pesticide use in animal and vegetable foods produced in Sweden. Sci. Total Environ..

[68] Ridoutt B., Navarro Garcia J. (2020). Cropland footprints from the perspective of productive land scarcity, malnutrition-related health impacts and biodiversity loss. J. Clean. Prod..

[69] Ridoutt B.G., Baird D., Hendrie G.A. (2021). Diets within planetary boundaries: What is the potential of dietary change alone?. Sustain. Prod. Consum..

[70] Fantke P., Chiu W.A., Aylward L., Judson R., Huang L., Jang S., Gouin T., Rhomberg L., Aurisano N., McKone T. (2021). Exposure and toxicity characterization of chemical emissions and chemicals in products: Global recommendations and implementation in USEtox. Int. J. Life Cycle Assess..

[71] Ernstoff A., Niero M., Muncke J., Trier X., Rosenbaum R.K., Hauschild M., Fantke P. (2019). Challenges of including human exposure to chemicals in food packaging as a new exposure pathway in life cycle impact assessment. Int. J. Life Cycle Assess..

