# Supplementary material for: Pesticide Toxicity Footprints of Australian Dietary Choices
Source: Nutrients. 2021 Nov 29;13(12):4314. doi: 10.3390/nu13124314 (PMC8703275; doi:10.3390/nu13124314)
Supplement: Supplementary file 1 [file nutrients-13-04314-s001.zip › nutrients-1460153-supplementary.pdf]

Supplementary Material

Suplementary Table S1 Normalization and weighting factors

**Normalization model***	**FE**	**HT-c**	**HT-nc**
Global	5.78×10^{13}	3.37×10^{5}	5.91×10^{6}
EU27	4.46×10^{12}	1.88×10^{4}	2.69×10^{5}
**Weighting model***	**FE**	**HT-c**	**HT-nc**
Equal	0.33	0.33	0.33
EU policy	0.32	0.36	0.32

* See main text for details

Supplementary Table S2 Environmental indicator values of individual foods

All data edible portion, uncooked	FE	HT-c	HT-nc	PTF
	CTUe/kg	CTUh/kg	CTUh/kg	pt/kg
**LIVESTOCK PRODUCTS OTHER THAN DAIRY**				
Egg	1.09	1.56×10^{-9}	7.04×10^{-9}	8.23
Chicken meat	2.41	2.28×10^{-9}	1.60×10^{-8}	17.08
Chicken offal	1.23	1.16×10^{-9}	8.13×10^{-9}	8.68
Pig meat	3.52	2.98×10^{-9}	2.94×10^{-8}	24.91
Pig offal	0.68	5.73×10^{-10}	5.66×10^{-9}	4.79
Lamb meat	1.89	1.15×10^{-9}	1.28×10^{-8}	12.78
Lamb offal	0.86	5.23×10^{-10}	5.80×10^{-9}	5.80
Beef meat	1.57	1.65×10^{-9}	9.54×10^{-9}	11.24
Beef offal	0.89	9.35×10^{-10}	5.42×10^{-9}	6.38
Game meat	0.00	0.00	0.00	0.00
Fish - wildcapture	0.00	0.00	0.00	0.00
Fish - farmed	0.67	1.42×10^{-9}	1.04×10^{-8}	5.85
Mussels	0.00	0.00	0.00	0.00
Prawn - wildcapture	0.00	0.00	0.00	0.00
Octopus/squid	0.00	0.00	0.00	0.00
Lobster	0.00	0.00	0.00	0.00
Beef sausage	1.26	1.35×10^{-9}	8.16×10^{-9}	9.04
Processed pig meat	3.52	2.98×10^{-9}	2.94×10^{-8}	24.91
Pork sausage	2.72	2.35×10^{-9}	2.31×10^{-8}	19.29
Lamb sausage	1.50	9.79×10^{-10}	1.06×10^{-8}	10.20
Processed chicken meat	2.41	2.28×10^{-9}	1.60×10^{-8}	17.08
Processed beef	1.57	1.65×10^{-9}	9.54×10^{-9}	11.24
Processed lamb	1.89	1.15×10^{-9}	1.28×10^{-8}	12.78
Chicken sausage	1.89	1.83×10^{-9}	1.30×10^{-8}	13.42
**DAIRY FOODS**				
Whole milk	0.31	2.98×10^{-10}	1.35×10^{-9}	2.14
Cheese	1.56	1.52×10^{-9}	6.88×10^{-9}	10.91
Butter	2.08	2.03×10^{-9}	9.18×10^{-9}	14.55
Cream	1.17	1.13×10^{-9}	5.13×10^{-9}	8.13
Skim milk	0.21	2.09×10^{-10}	9.45×10^{-10}	1.50
Yogurt (sugar, no fruit)	0.40	3.08×10^{-10}	1.40×10^{-9}	2.66
Yogurt (no sugar, no fruit)	0.34	3.28×10^{-10}	1.48×10^{-9}	2.35
Ice-cream (sugar, plain)	0.65	4.66×10^{-10}	2.12×10^{-9}	4.34
Milk, chocolate flavour	0.45	2.81×10^{-10}	1.28×10^{-9}	2.96
Milk, iced coffee flavour	0.43	2.82×10^{-10}	1.28×10^{-9}	2.85
Milk, other flavour	0.36	2.83×10^{-10}	1.28×10^{-9}	2.41
Milk powder	2.39	2.32×10^{-9}	1.05×10^{-8}	16.68
Condensed milk	1.14	4.08×10^{-10}	1.87×10^{-9}	7.09
Custard	0.42	3.69×10^{-10}	1.32×10^{-9}	2.89
**SWEETENERS**				
Sugar-refined	1.30	4.31×10^{-12}	5.80×10^{-11}	7.52

Molasses	0.27	8.88×10^{-13}	1.19×10^{-11}	1.55
Honey	0.00	0.00	0.00	0.00
**VEGETABLES**				
Potato	0.37	2.93×10^{-10}	9.11×10^{-10}	2.46
Onion	0.39	3.11×10^{-10}	9.68×10^{-10}	2.62
Garlic	0.38	3.00×10^{-10}	9.33×10^{-10}	2.52
Beetroot	0.45	3.61×10^{-10}	1.12×10^{-9}	3.03
Carrot	0.41	3.23×10^{-10}	1.01×10^{-9}	2.72
Swede	0.35	2.80×10^{-10}	8.70×10^{-10}	2.35
Tomato salad (passive production system)	0.32	2.52×10^{-10}	7.82×10^{-10}	2.12
Celery	0.48	3.83×10^{-10}	1.19×10^{-9}	3.22
Zucchini	0.34	2.68×10^{-10}	8.32×10^{-10}	2.25
Squash	0.33	2.62×10^{-10}	8.15×10^{-10}	2.20
Cucumber	0.32	2.57×10^{-10}	7.98×10^{-10}	2.16
Pumpkin	0.39	3.11×10^{-10}	9.68×10^{-10}	2.62
Mushroom	0.31	2.49×10^{-10}	7.74×10^{-10}	2.09
Beans - green	0.39	3.11×10^{-10}	9.68×10^{-10}	2.62
Cauliflower	0.52	4.15×10^{-10}	1.29×10^{-9}	3.49
Broccoli	0.52	4.15×10^{-10}	1.29×10^{-9}	3.49
Cabbage	0.39	3.11×10^{-10}	9.68×10^{-10}	2.62
Snowpea	0.39	3.11×10^{-10}	9.68×10^{-10}	2.62
Peas (no pod)	0.49	3.89×10^{-10}	1.21×10^{-9}	3.27
Spinach and other leafy greens	0.42	3.36×10^{-10}	1.05×10^{-9}	2.83
Lettuce and other salad leaves	0.45	3.56×10^{-10}	1.11×10^{-9}	2.99
Ginger	0.33	2.65×10^{-10}	8.23×10^{-10}	2.23
Asparagus	0.38	3.00×10^{-10}	9.33×10^{-10}	2.52
Capsicum and peppers	0.39	3.11×10^{-10}	9.68×10^{-10}	2.62
Avocado	30.64	4.24×10^{-10}	1.29×10^{-9}	177.21
Sweet corn	0.60	4.79×10^{-10}	1.49×10^{-9}	4.03
Fennel	0.56	4.45×10^{-10}	1.38×10^{-9}	3.74
**FRUITS**				
Orange, fresh	13.64	8.68×10^{-10}	5.82×10^{-9}	79.85
Lemon, fresh	15.91	1.01×10^{-9}	6.79×10^{-9}	93.16
Lime, fresh	15.00	9.54×10^{-10}	6.40×10^{-9}	87.83
Grapefruit, fresh	15.22	9.68×10^{-10}	6.49×10^{-9}	89.11
Mandarin, fresh	13.82	8.79×10^{-10}	5.89×10^{-9}	80.90
Apple, fresh	0.53	1.04×10^{-10}	5.85×10^{-9}	3.51
Pear, fresh	0.56	1.09×10^{-10}	6.11×10^{-9}	3.67
Quince, fresh	0.57	1.12×10^{-10}	6.26×10^{-9}	3.75
Grape, fresh	2.29	1.72×10^{-11}	1.68×10^{-8}	14.15
Watermelon, fresh	0.61	4.88×10^{-10}	1.52×10^{-9}	4.11
Rockmelon, fresh	0.50	3.95×10^{-10}	1.23×10^{-9}	3.32
Honey dew melon, fresh	0.50	4.02×10^{-10}	1.25×10^{-9}	3.38
Apricot, fresh	7.28	3.43×10^{-10}	9.68×10^{-8}	47.78
Peach, fresh	7.60	3.58×10^{-10}	1.01×10^{-7}	49.90
Nectarine, fresh	7.44	3.50×10^{-10}	9.89×10^{-8}	48.82

Cherry, fresh	8.05	3.79×10^{-10}	1.07×10^{-7}	52.84
Kiwi, fresh	9.12	4.29×10^{-10}	1.21×10^{-7}	59.88
Pineapple, fresh	4.88	1.51×10^{-10}	1.43×10^{-10}	28.29
Banana, fresh	5.03	1.55×10^{-10}	1.48×10^{-10}	29.16
Strawberry, fresh	7.05	3.32×10^{-10}	9.38×10^{-8}	46.30
Raspberry, fresh	6.84	3.22×10^{-10}	9.10×10^{-8}	44.91
Cranberry, fresh	6.84	3.22×10^{-10}	9.10×10^{-8}	44.91
Blueberry, fresh	6.84	3.22×10^{-10}	9.10×10^{-8}	44.91
Olive, fresh	8.55	4.03×10^{-10}	1.14×10^{-7}	56.14
Mango, fresh	32.45	4.49×10^{-10}	1.36×10^{-9}	187.63
**VEGETABLE OILS**				
Canola oil	4.77	3.04×10^{-9}	1.95×10^{-8}	31.60
Soybean oil	1.30	2.51×10^{-9}	1.32×10^{-9}	10.04
Sesame oil	9.38	3.30×10^{-9}	4.00×10^{-8}	59.64
Olive oil	32.84	1.55×10^{-9}	4.37×10^{-7}	215.57
Peanut oil	1.25	2.41×10^{-9}	1.26×10^{-9}	9.64
Sunflower oil	7.97	2.81×10^{-9}	3.40×10^{-8}	50.67
Palm oil	20.57	2.44×10^{-9}	1.09×10^{-8}	121.64
**NUTS**				
Other nuts	39.33	6.17×10^{-9}	1.50×10^{-7}	241.41
Almond	60.45	9.48×10^{-9}	2.31×10^{-7}	371.02
Peanut	0.94	1.82×10^{-9}	9.55×10^{-10}	7.28
**SEEDS**				
Sesame seed	4.04	1.42×10^{-9}	1.72×10^{-8}	25.65
Other seeds	4.04	1.42×10^{-9}	1.72×10^{-8}	25.65
**GRAINS**				
Brown rice	0.06	1.99×10^{-10}	1.55×10^{-8}	1.44
White rice	0.07	2.27×10^{-10}	1.77×10^{-8}	1.64
Rice flour	0.07	2.27×10^{-10}	1.77×10^{-8}	1.64
Rice bran	0.04	1.13×10^{-10}	8.86×10^{-9}	0.82
Barley	0.26	4.93×10^{-10}	4.85×10^{-9}	2.24
Corn flour	1.46	2.07×10^{-9}	2.36×10^{-9}	10.61
Oats	0.26	5.08×10^{-10}	4.99×10^{-9}	2.30
Wholemeal wheat flour	0.22	4.20×10^{-10}	4.13×10^{-9}	1.91
Refined wheat flour	0.20	3.92×10^{-10}	3.85×10^{-9}	1.78
Wheat bran	0.27	5.10×10^{-10}	5.01×10^{-9}	2.32
Wheat germ	0.55	1.06×10^{-9}	1.04×10^{-8}	4.81
**PULSES/BEANS**				
Chickpea soaked	1.72	1.74×10^{-10}	1.29×10^{-8}	10.82
Lentil soaked	1.72	1.74×10^{-10}	1.29×10^{-8}	10.82
Tofu	0.14	2.80×10^{-10}	1.47×10^{-10}	1.12
**TEA & COFFEE**				

Tea (dried)	0.51	8.31×10^{-13}	2.93×10^{-10}	2.95
Coffee bean (roasted)	15.50	1.20×10^{-10}	3.48×10^{-10}	89.52
Coffee instant	31.00	2.40×10^{-10}	6.95×10^{-10}	179.04
**COCOA PRODUCTS**				
Cocoa powder	9.97	6.54×10^{-11}	3.87×10^{-10}	57.57
Cocoa paste	18.87	1.24×10^{-10}	7.33×10^{-10}	108.96
Milk Chocolate	6.84	5.99×10^{-10}	2.78×10^{-9}	40.17
**SPREADS**				
Margarine	3.58	1.94×10^{-9}	1.49×10^{-8}	23.40
Hazelnut spread	8.40	1.12×10^{-9}	5.46×10^{-9}	49.84
Jam-strawberry	3.34	1.35×10^{-10}	3.75×10^{-8}	21.53
Jam-raspberry	3.26	1.31×10^{-10}	3.64×10^{-8}	20.97
Jam -apricot	3.43	1.39×10^{-10}	3.87×10^{-8}	22.12
**DRIED FRUIT**				
Sultata	10.75	8.09×10^{-11}	7.90×10^{-8}	66.51
Dried apricot	37.85	1.78×10^{-9}	5.03×10^{-7}	248.44
**PROCESSED FRUITS**				
Pear, processed	0.56	1.09×10^{-10}	6.11×10^{-9}	3.67
Apricot, processed	7.28	3.43×10^{-10}	9.68×10^{-8}	47.78
Peach, processed	7.60	3.58×10^{-10}	1.01×10^{-7}	49.90
Pineapple, processed	4.88	1.51×10^{-10}	1.43×10^{-10}	28.29
Blueberry, processed	6.84	3.22×10^{-10}	9.10×10^{-8}	44.91
Raspberry, processed	6.84	3.22×10^{-10}	9.10×10^{-8}	44.91
**FRUIT JUICE**				
Grape juice	2.95	2.22×10^{-11}	2.17×10^{-8}	18.26
Orange juice	18.90	1.20×10^{-9}	8.06×10^{-9}	110.67
Apple juice	0.76	1.48×10^{-10}	8.29×10^{-9}	4.97
Blackcurrant juice	13.68	6.44×10^{-10}	1.82×10^{-7}	89.82
Pineapple juice	16.34	5.05×10^{-10}	4.80×10^{-10}	94.78
Lemon juice	26.26	1.67×10^{-9}	1.12×10^{-8}	153.71
Grapefruit juice	21.01	1.34×10^{-9}	8.96×10^{-9}	122.97
**COCONUT PRODUCTS**				
Coconut dessicated	53.49	6.34×10^{-9}	2.84×10^{-8}	316.33
Coconut water	11.72	1.39×10^{-9}	6.22×10^{-9}	69.32
Coconut milk	12.21	1.45×10^{-9}	6.48×10^{-9}	72.21
Coconut cream	24.42	2.90×10^{-9}	1.30×10^{-8}	144.42
**PROCESSED VEGETABLES**				
Processed potato	0.37	2.93×10^{-10}	9.11×10^{-10}	2.46
Processed beetroot	0.45	3.61×10^{-10}	1.12×10^{-9}	3.03
Processed tomato whole/diced	0.32	2.52×10^{-10}	7.82×10^{-10}	2.12
Processed mushroom	0.31	2.49×10^{-10}	7.74×10^{-10}	2.09

Processed beans-green	0.39	3.11×10^{-10}	9.68×10^{-10}	2.62
Processed pea (no pods)	0.49	3.89×10^{-10}	1.21×10^{-9}	3.27
Processed spinach	0.42	3.36×10^{-10}	1.05×10^{-9}	2.83
Processed asparagus	0.38	3.00×10^{-10}	9.33×10^{-10}	2.52
Processed corn kernal	0.60	4.79×10^{-10}	1.49×10^{-9}	4.03
**VEGETABLE JUICE/PUREE/PASTE**				
Vegetable juice	0.63	4.98×10^{-10}	1.55×10^{-9}	4.19
Tomato juice	0.39	3.11×10^{-10}	9.68×10^{-10}	2.62
Tomato puree	0.39	3.11×10^{-10}	9.68×10^{-10}	2.62
Tomato paste	1.25	9.96×10^{-10}	3.10×10^{-9}	8.38
**SAUCES/DRESSINGS**				
Balsamic vinegar	0.29	2.22×10^{-12}	2.17×10^{-9}	1.83
Soy sauce	0.14	2.66×10^{-10}	1.39×10^{-10}	1.06
Mayonnaise	5.37	1.90×10^{-9}	2.24×10^{-8}	34.10
Tomato sauce	0.59	2.44×10^{-10}	7.67×10^{-10}	3.70
Barbecue sauce	0.80	1.73×10^{-10}	5.58×10^{-10}	4.83
**BAKED FOODS**				
White bread	0.14	2.74×10^{-10}	2.70×10^{-9}	1.25
Wholemeal bread	0.15	2.94×10^{-10}	2.89×10^{-9}	1.34
Crispbread-wholegrain	0.67	5.83×10^{-10}	5.46×10^{-9}	4.73
Crispbread-plain	0.50	5.53×10^{-10}	4.75×10^{-9}	3.72
Biscuit-sweet-plain	0.95	5.94×10^{-10}	4.68×10^{-9}	6.36
Biscuit-choc crème and coated	3.50	6.77×10^{-10}	4.30×10^{-9}	21.11
Croissant	0.67	7.48×10^{-10}	4.92×10^{-9}	4.90
Cake -Madiera type	1.31	8.72×10^{-10}	5.47×10^{-9}	8.71
Crème biscuit	1.57	7.10×10^{-10}	5.10×10^{-9}	10.06
Oat biscuit	5.17	1.09×10^{-9}	6.30×10^{-9}	31.27
Cone for ice-cream	0.97	6.99×10^{-10}	4.07×10^{-9}	6.53
Water cracker	0.75	7.09×10^{-10}	5.73×10^{-9}	5.36
Rice cracker	0.21	3.11×10^{-10}	1.78×10^{-8}	2.54
Corn cake	1.50	2.08×10^{-9}	2.53×10^{-9}	10.82
Museli bar: nuts & seeds	5.08	1.50×10^{-9}	2.20×10^{-8}	32.01
Museli Bar: fruit & nut	10.31	1.61×10^{-9}	9.15×10^{-8}	66.21
Museli bar: nuts & seeds/choc coat	5.43	1.32×10^{-9}	1.81×10^{-8}	33.64
Iced doughnut	1.04	6.82×10^{-10}	5.09×10^{-9}	6.94
Cake, fruit, commercial, uniced	4.70	8.06×10^{-10}	3.25×10^{-8}	29.74
Cake, lamington, unfilled	5.82	1.08×10^{-9}	5.88×10^{-9}	34.95
Cake or cupcake, chocolate, commercial, sugar based icing	1.77	6.46×10^{-10}	4.02×10^{-9}	11.05
Muffin, cake-style, berry, commercial, uniced	2.26	9.10×10^{-10}	1.81×10^{-8}	14.96
**PASTA**				
Pasta	0.20	3.92×10^{-10}	3.85×10^{-9}	1.78
**BEVERAGES**				

Tap water	0.00	0.00	0.00	0.00
Bottled water	0.00	0.00	0.00	0.00
Soft drink (sugar sweetened)	0.14	4.75×10^{-13}	6.38×10^{-12}	0.83
Sports drink	0.09	3.02×10^{-13}	4.06×10^{-12}	0.53
Soft drink (artifical sweetener)	0.00	0.00	0.00	0.00
Rice beverage	0.03	4.70×10^{-11}	2.58×10^{-9}	0.39
Almond beverage	0.58	1.02×10^{-10}	2.22×10^{-9}	3.56
Soy beverage	0.08	9.29×10^{-11}	2.28×10^{-10}	0.57
Chocolate and malt powder (as powder)	2.50	7.65×10^{-10}	6.98×10^{-9}	15.57
Cordial - conc - not juice based	0.52	1.73×10^{-12}	2.33×10^{-11}	3.02
Cordial - conc - juice based	6.60	3.23×10^{-10}	1.81×10^{-9}	38.50
**SNACKS**				
Popcorn	2.93	2.32×10^{-9}	9.68×10^{-9}	19.77
Extruded cheese snack	1.80	1.66×10^{-9}	1.22×10^{-8}	12.70
Potato chips (crisps)	3.09	2.17×10^{-9}	1.06×10^{-8}	20.57
Cornchips	2.35	2.33×10^{-9}	6.98×10^{-9}	16.26
**CONFECTIONARY**				
Lolly (jube type)	0.72	5.81×10^{-10}	1.34×10^{-9}	4.81
Licorice	0.56	1.19×10^{-10}	1.18×10^{-9}	3.40
**BREAKFAST CEREALS**				
Muesli, natural with dried fruit (nut free)	5.08	6.09×10^{-10}	6.24×10^{-8}	33.42
Wheat biscuit	0.24	3.94×10^{-10}	3.87×10^{-9}	2.01
Oat cluster fruit	1.17	6.18×10^{-10}	8.37×10^{-9}	7.84
Oat cluster nut	4.12	1.17×10^{-9}	1.78×10^{-8}	25.93
Puffed rice	0.17	1.81×10^{-10}	1.41×10^{-8}	1.98
Cornflake	1.94	2.57×10^{-9}	2.94×10^{-9}	13.88
Puffed rice with chocolate	0.88	6.20×10^{-12}	1.92×10^{-10}	5.10
Cornflake, frosted	1.70	1.61×10^{-9}	1.86×10^{-9}	11.50
Commercial mixed grain/dried fruit/sugar	1.60	8.04×10^{-10}	9.25×10^{-9}	10.55
**POTATO CHIPS - FRENCH FRIES**				
Potato chips - oven fry - basic	0.57	4.25×10^{-10}	1.76×10^{-9}	3.81
Potato chips - oven fry - coated	0.61	4.94×10^{-10}	1.81×10^{-9}	4.13
**PIES & PASTRIES**				
Pastry	0.97	7.26×10^{-10}	5.66×10^{-9}	6.61
Meat pie	0.94	8.58×10^{-10}	5.31×10^{-9}	6.54
Apple pie	0.98	3.32×10^{-10}	5.49×10^{-9}	6.26
Quiche lorrianne	1.31	1.24×10^{-9}	8.19×10^{-9}	9.24
Danish Custard	0.79	6.14×10^{-10}	3.79×10^{-9}	5.38
Pasty, filled with vegetables & meat	0.92	7.15×10^{-10}	4.53×10^{-9}	6.26
**ALCOHOLIC BEVERAGES**				
Wine	3.19	2.40×10^{-11}	2.34×10^{-8}	19.73
Beer	0.07	1.28×10^{-10}	1.25×10^{-9}	0.58

Apple cider	0.76	1.48 x 10^{-10}	8.29 x 10^{-9}	4.97
Spirit (rum)	0.29	9.68 x 10^{-13}	1.30 x 10^{-11}	1.69

Freshwater ecotoxicity (FE); Human toxicity carcinogenic effects (HT-c), Human toxicity non-carcinogenic effects (HT-nc), Pesticide toxicity footprint (PTF)

Supplementary Figure S1 Environmental indicator results for Australian adult daily diets

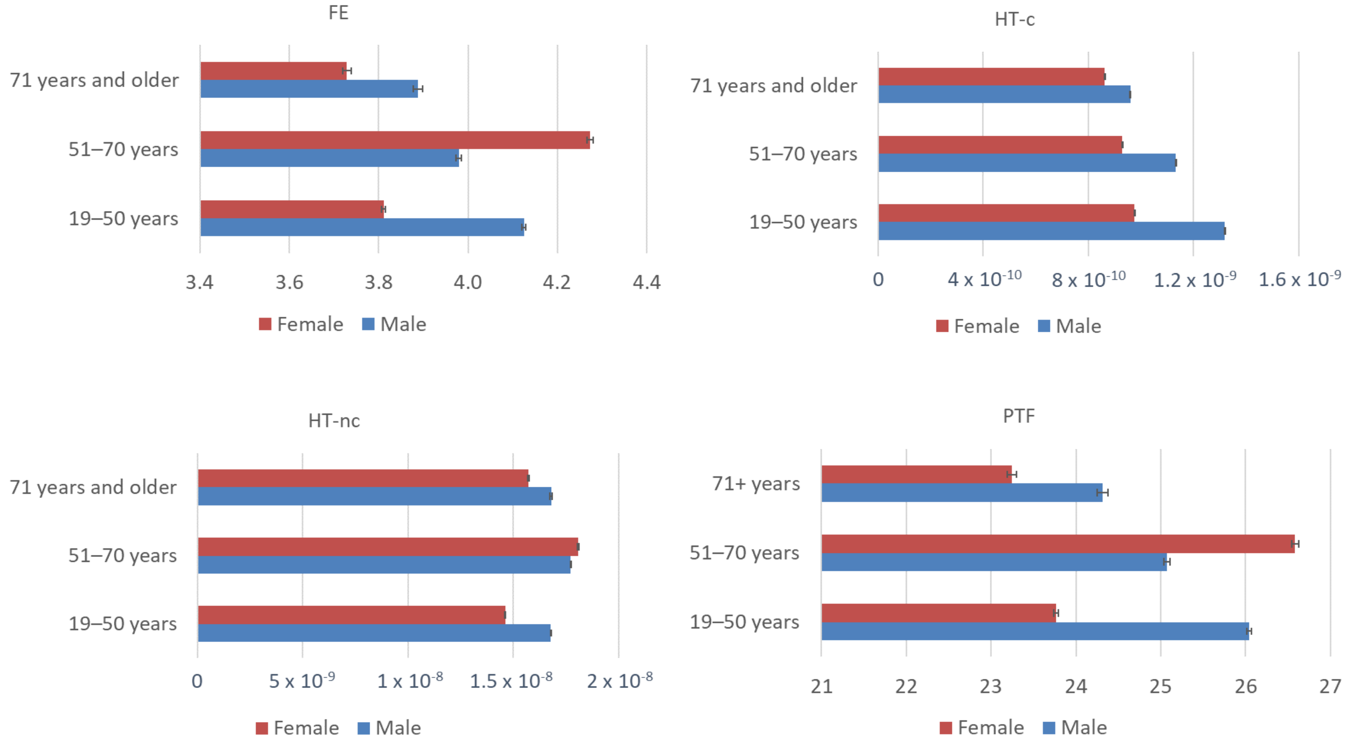

Freshwater ecotoxicity (FE, CTUe/person/day); Human toxicity carcinogenic effects (HT-c, CTUh/person/day), Human toxicity non-carcinogenic effects (HT-nc, CTUh/person/day), Pesticide toxicity footprint (PTF, points/person/day)
